# Silica nanoparticles functionalized via click chemistry and ATRP for enrichment of Pb(II) ion

**DOI:** 10.1186/1556-276X-7-485

**Published:** 2012-08-29

**Authors:** Wei Li, Yaohui Xu, Yang Zhou, Wenhui Ma, Shixing Wang, Yongnian Dai

**Affiliations:** 1 Faculty of Metallurgical and Energy Engineering, Kunming University of Science and Technology, Kunming, 650093, China; 2 National Engineering Laboratory for Vacuum Metallurgy, Kunming University of Science and Technology, Kunming, 650093, China

**Keywords:** Silica nanoparticles, Surface modification, Click, ATRP, Enrichment, Pb(II)

## Abstract

Silica nanoparticles have been functionalized by click chemistry and atom transfer radical polymerization (ATRP) simultaneously. First, the silanized silica nanoparticles were modified with bromine end group, and then the azide group was grafted onto the surface via covalent coupling. 3-Bromopropyl propiolate was synthesized, and then the synthesized materials were used to react with azide-modified silica nanoparticles via copper-mediated click chemistry and bromine surface-initiated ATRP. Transmission electron microscopy, Fourier transform infrared spectroscopy, X-ray photoelectron spectroscopy, and thermogravimetric analysis were performed to characterize the functionalized silica nanoparticles. We investigated the enrichment efficiency of bare silica and poly(ethylene glycol) methacrylate (PEGMA)-functionalized silica nanoparticles in Pb(II) aqueous solution. The results demonstrated that PEGMA-functionalized silica nanoparticles can enrich Pb(II) more quickly than pristine silica nanoparticles within 1 h.

## Background

In recent years, silica nanoparticles (SNPs) have received significant attention due to their chemical inertness, nontoxicity, optical transparency, and excellent thermal stability [1-3], which can be widely used in catalysis [4], chemical process industry [5], removal of metal ions [6], and metal ion preconcentration [7-9] through polymer coatings or other functional groups. For many applications, there are several chemical methods for controlling the nanoparticles’ surface functionality, such as chemisorptions [10], sol–gel process, and immobilization of organic molecules by silane coupling reagents [11], which can result in the immobility, mechanical stability, and water insolubility of functionalized SNPs [12]. Polymerization methods involving living free radical nitroxide-mediated polymerization, reversible addition-fragmentation chain transfer (RAFT), and atom transfer radical polymerization (ATRP) were commonly considered as effective techniques to functionalize materials [13-16]. Especially, the ATRP technique has been developed rapidly due to the advantages of being simple, inexpensive, and more general for controlled radical polymerization when it was firstly proposed by Wang and Matyjaszewsi [17]. There are a greater number of researchers using ATRP to functionalize SNPs [18-22].

With the appearance of the click chemistry proposed by Sharpless [23], the copper(I)-catalyzed azide-alkyne 1,3-dipolar cycloaddition reaction also becomes a particularly powerful approach to synthesize designed molecules or modify inorganic materials because only mild reaction conditions are required and the extreme selectivity toward molecules bearing azides and alkynes prevents unwanted side product [24-26]. Recently, some literatures reported that gold nanoparticles, silicon oxides, carbon nanotubes, and other materials were functionalized by click chemistry [27-30].

Based on the advantages of ATRP and click chemistry, the combination of these two methods has attracted attention to functionalize nanomaterials. Ranjan and Brittain [31] successfully grafted polymer chains onto SNPs through a combination of RAFT polymerization and click chemistry. Wang and co-workers [32] utilized ATRP and click chemistry to modify particles. Polystyrene brushes were firstly introduced on the surface via ATRP, and then the click reaction was generated on the surface of polymerized SNPs. The combination of surface-initiated ATRP and Huisgen [3 + 2] cycloaddition [33,34] was also developed as a versatile method for the functionalizations of SNPs. Most of the literatures reported the combination of ATRP and click chemistry to modify nanomaterials in tandem.

**Figure 1:**
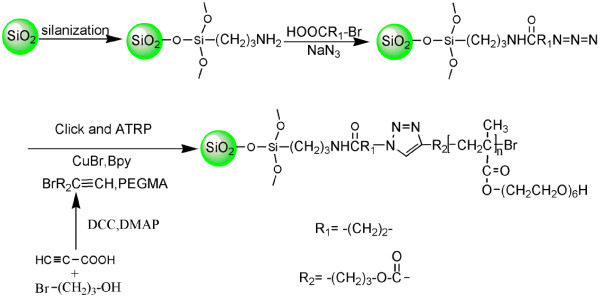
The steps of functionalized silica nanoparticles by click chemistry and ATRP.

Herein, we described the immobilization of the initiator on the SNP surface via 1,3-dipolar cycloaddition, which can simultaneously initiate surface-induced ATRP of poly(ethylene glycol) methacrylate on the SNP surface. The general steps for the preparation of functionalized SNPs are shown in Figure 1. Based on the special characteristics of the ethylene glycol end group, we investigated the enrichment properties of Pb(II) in aqueous solution.

## Methods

### Materials

3-Bromopropanoic acid and propargyl acid were obtained from Alfa Aesar (Ward Hill, MA, USA). Poly(ethylene glycol) methacrylate (PEGMA), 1,3-dicyclohexylcarbodiimide (DCC), *N*-hydroxysuccinimide (NHS), 1-ethyl-3-(3-dimethylaminopropyl)carbodiimide hydrochloride (EDC), 4-dimethylaminopyridime (DMAP), and sodium azide (NaN_3_) were obtained from Sigma-Aldrich Corporation (St. Louis, MO, USA). 3-Aminopropyltriethoxysilane (APTES) was purchased from Dow Corning Corporation (Midland, MI, USA). CuBr and 3-bromo-1-propanol were obtained from Shanghai Jingchun Reagent Co., Ltd. (Aladdin; Shanghai, China). Tetraethyl orthosilicate (TEOS) and 2,2^′^-bypiridine (Bpy) were purchased from Tianjin Chemical Reagent Co. Ltd. (Tianjin, China).

### Synthesis of 3-bromopropyl propiolate

3-Bromo-1-propanol (3 ml, 33 mmol), DCC (5 g, 25.5 mmol), and DMAP (0.6 g, 5.04 mmol) were dissolved in 100 ml of dichloromethane (DCM), then propargyl acid (4.5 ml, 42 mmol) was added into the solution slowly. The mixture was stirred at room temperature in a dark room for 24 h, diluted with DCM, filtered off, and washed with DCM until white particles were observed. Then, the liquid was evaporated completely using a rotary evaporator. The products were dried in a clean vacuum at 50°C overnight.

### Preparation and modification of silica particles

The spherical silica particles were prepared from TEOS using NH_3_·H_2_O as catalyst according to the Stöber method [35]. Then, 65 ml of ethanol, 3 ml of deionized water, and 10 ml of NH_3_·H_2_O (25%) were added into a flask with violent stirring at 40°C for 2 h; 5 ml of TEOS was added into the above solution dropwise. The mixture was kept at 40°C with stirring overnight. After the reaction, the mixture was separated by centrifugation and the white particles were washed with water, ethanol, and toluene, respectively. Then, the silica particles were dispersed in 50 ml of toluene by ultrasonication to produce a homogeneous suspension.

The surface of SiO_2_ has functioned as an amino group by a silanization reaction according to a previous report. Namely, the prepared SiO_2_ homogeneous suspension was put into a 100-ml round flask, and APTES (5.3 ml, 23 mmol) was added using a syringe. The reaction mixture was refluxed at 95°C in oil bath with stirring for 10 h. The obtained amino group-immobilized silica particles, defined as SiO_2_-NH_2_, were collected by centrifugation and washed with ethanol (2 × 50 ml) and *N*,*N*-dimethylformamide (DMF; 2 × 50 ml) in turn, then dispersed in 50 ml of DMF to immobilize the azide group.

3-Bromopropionic acid (2.8 g, 18.4 mmol), NHS (2.4 g, 7 mmol), and EDC (4 g, 22.3 mmol) were added to the SiO_2_-NH_2_ and DMF solution with stirring and protected from light at 80°C under N_2_. After 24 h, 3 g of NaN_3_ was added into the above system for 24 h. Then, particles called SiO_2_-N_3_ were collected by centrifugation and washed with water and DMF.

### Surface-initiated atom transfer radical polymerization by click chemistry

The reaction involved in click reaction and atom transfer radical polymerization was described as follows: The prepared azide-modified SiO_2_ and 3-bromopropyl propiolate were dispersed in 50 ml of DMF, then CuBr (43 mg, 0.3 mmol), Bpy ligand (90 mg, 0.6 mmol), and PEGMA (10 ml, 30 mmol) were added quickly; the reaction was stirred at 80°C under N_2_ for 24 h. After the reaction, the grafted PEGMA silica particles were collected by centrifugation, referred as SiO_2_-PEGMA; the product was washed with DCM at least three times to remove the excess reactant, then washed with ethanol and deionized water, and dried in a clean vacuum oven at 50°C overnight.

### Sensing of Pb^2+^

Sensing of Pb^2+^ experiments were carried out by the following steps: a 10 ml portion of the aqueous sample solution, containing 100 mg/l Pb^2+^, was prepared, and the pH value was adjusted to 6 with aqueous HCl. Then, 50 mg of SiO_2_ nanoparticles and SiO_2_-PEGMA were dispersed in eight portions of the above solution with an ultrasonic oscillator, and the solutions were stewed for 0.5, 1, 2, and 3 h, respectively. The particles were separated by centrifugation. The supernatant was detected by atomic absorption spectroscopy (AAS).

### Surface characterization

Fourier transform infrared (FT-IR) spectra were obtained using a Spectrum One FT-IR spectrometer (IR Prestige-21, Shimadzu Corporation, Kyoto, Japan) with a resolution of 4 cm^−1^. To characterize the layers formed on the surface of the nanoparticle, the powder was milled with KBr, and the mixture was pressed into a disk for analysis. The morphology and size of SNPs were studied by transmission electron microscopy (TEM) with a JEOL 200CX (Akishima-shi, Japan). X-ray photoelectron spectroscopy (XPS) was performed on a PHI 5500 electron spectrometer (Physical Electronics, Inc., Chanhassen, MN, USA) using 200-W Mg radiations. The binding energies were referenced to the C 1*s* line at 284.8 eV from adventitious carbon. Thermogravimetric analysis (TGA) was carried out using a Netzsch STA449 F3 thermogravimetric instrument (Wolverhampton, UK) at a heating rate of 10°C/min under a flow of nitrogen. AAS was carried out using an AA-6800 instrument (Shimadzu Corporation).

## Results and discussion

**Figure 2:**
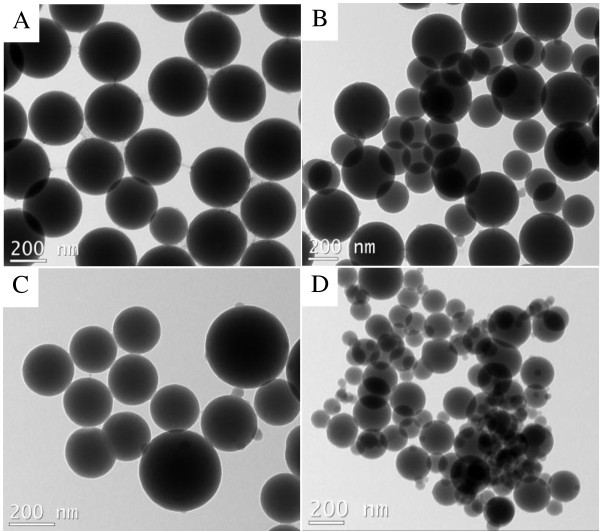
**TEM images.** (**A**) Silica nanoparticles, (**B**) silica nanoparticles treated with APTES, (**C**) azide-functionalized silica nanoparticles, and (**D**) silica nanoparticles modified via click chemistry and ATRP simultaneously.

We performed TEM to characterize the morphologies of SiO_2_ and modified SiO_2_ nanoparticles (Figure 2). From Figure 2A, we can see clearly that the mean size of the particles is about 300 nm and the shape shows regulated sphericity. The average diameter of amino group-modified SNPs is about 355 nm (Figure 2B). Azide-functionalized SNPs and SNPs modified via click chemistry and ATRP simultaneously are presented in Figure 2C,D, respectively. The shape of the modified SNPs maintained the original spherical morphology.

**Figure 3:**
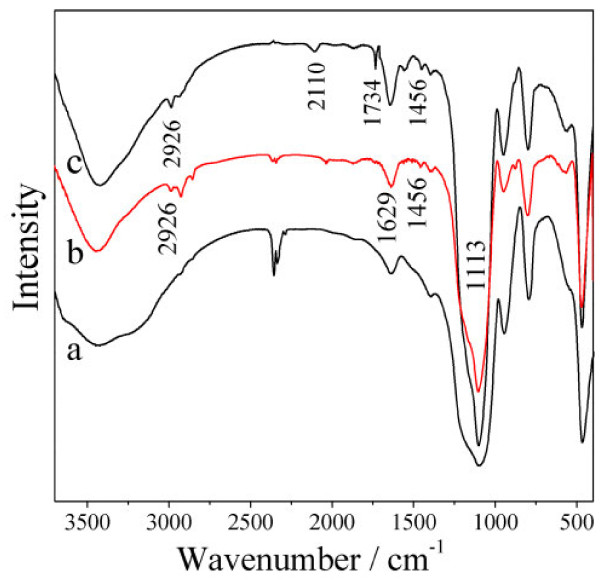
**FT-IR spectra of SiO**_**2**_**(a), SiO**_**2**_**-NH**_**2**_**(b), and SiO**_**2**_**-N**_**3**_**(c) nanoparticles.**

The FT-IR spectra of SiO_2_, SiO_2_-NH_2_, and SiO_2_-N_3_ are shown in Figure 3. Strong adsorption peaks at about 1,113 cm^−1^ were observed for all the samples, indicating the existence of Si-O-Si stretching vibration of silanol groups. The -CH_2_ groups were confirmed by C-H stretching at 2,926 cm^−1^ and C-H scissoring vibration at 1,456 cm^−1^. O-H stretching and bending vibrations could be detected at 1,629 and 3,600 to 3,300 cm^−1^. In Figure 3b, the adsorption peak at about 3,420 cm^−1^ was assigned to N-H stretching vibration, suggesting that the amino organic groups were introduced to the surface of SNPs. A peak at 1,734 cm^−1^ observed in Figure 3c corresponded to the C = O stretching. Furthermore, the azide group-modified SNPs are an essential intermediate for the click chemistry reaction, which was demonstrated by a new peak at 2,110 cm^−1^ in the spectrum of Figure 3c.

**Figure 4:**
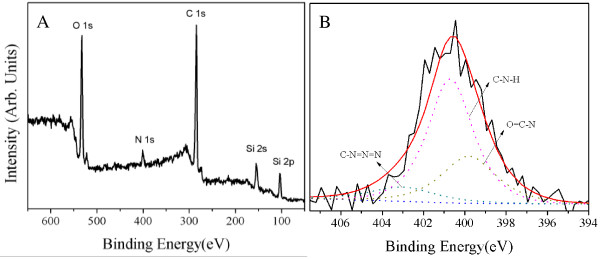
**Wide-scan (A) and N 1*s*core-level (B) spectra of azide-functionalized silica nanoparticles.**

In order to further confirm the SNPs modified via click chemistry and ATRP simultaneously, the products were characterized by XPS analysis. The introduction of the azide group onto SNPs was demonstrated by the XPS spectrum in Figure 4, which presented the wide-scan (A) and N 1*s* core-level (B) spectra. The wide-scan spectra of the SiO_2_-N_3_ surface was dominated clearly by signals attributable to Si 2*p*, Si 2*s*, C 1*s*, N 1*s*, and O 1*s* with binding energies at about 101.2, 154, 284.2, 401.2, and 535.2 eV, respectively. The N 1*s* core-level spectra of azide-modified SNPs can be curve-fitted into three peak components with binding energies at about 399.8, 400.7, and 403 eV, attributable to the O = C-N, C-N-H, and C-N = N = N species, respectively.

**Figure 5:**
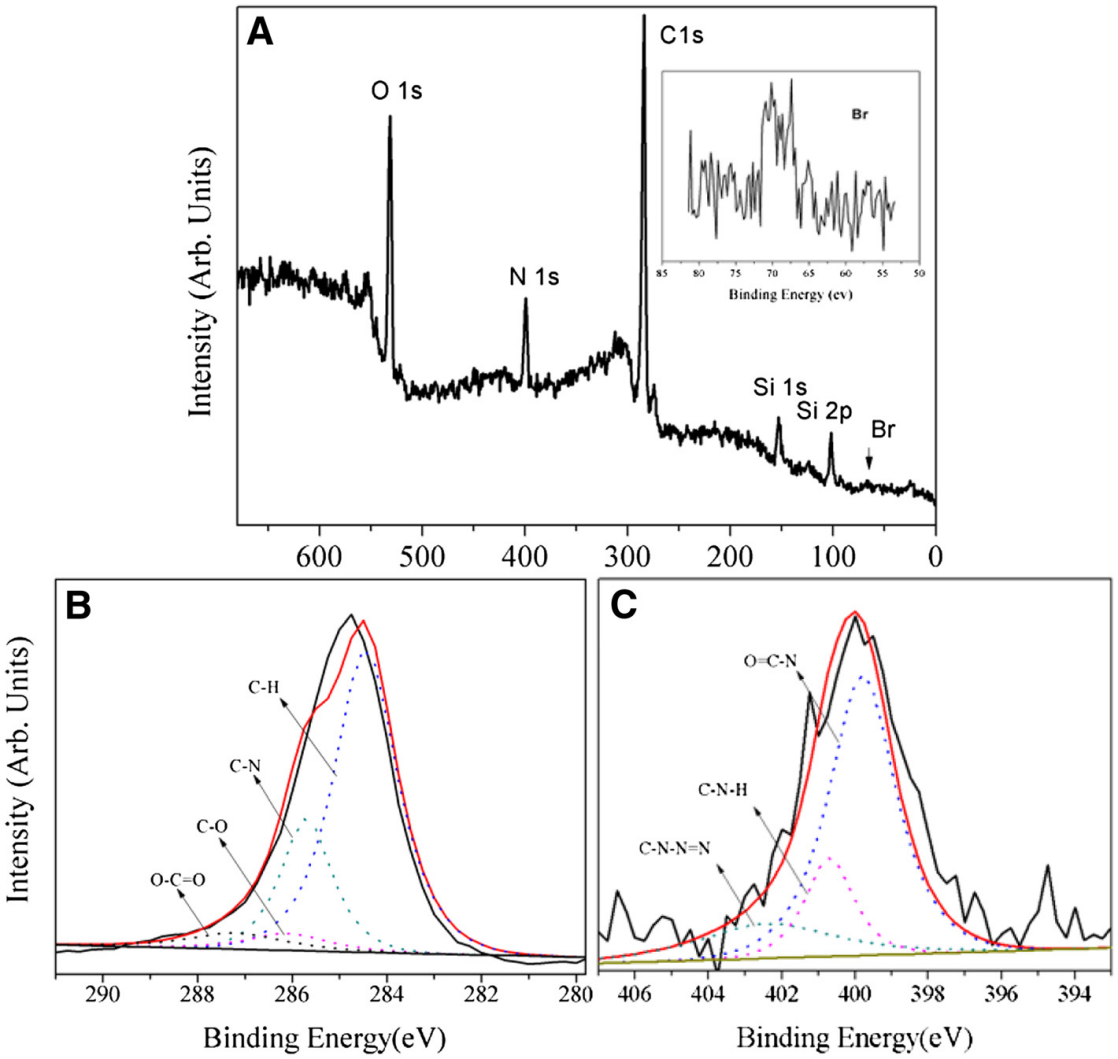
**Wide-scan (A) and C 1*s*(B) and N 1*s*(C) core-level spectra of modified SNPs.** The inset in (A) is Br 3*d* core-level spectrum.

Figure 5A showed the wide-scan spectrum of the SiO_2_-PEGMA surface, which was dominated by signals attributable to Si 2*p*, Si 2*s*, C 1*s*, N 1*s*, O 1*s*, and Br with binding energies at about 101.8, 153, 284.2, 399.4, 531.4, and 67.4 eV, respectively. The peak of Br was observed at 67.4 eV, which could have originated from 3-bromopropyl propiolate. Figure 5B and C present the XPS spectra of C 1*s* and N 1*s* narrow scan on the SiO_2_-PEGMA nanoparticles. The C 1*s* core-level spectrum can be curve-fitted with four peak components having binding energies at about 284.4, 285.7, 286.2, and 287.2 eV attributable to the C-H, C-N, C-O, and O = C-O species, respectively. The N 1*s* core-level spectrum can be curve-fitted into three peak components with binding energies at about 397.9, 399.8, and 400.7 eV which are ascribed to the C-N-N = N, O = C-N, and C-N-H species, respectively (Figure 5C).

**Figure 6:**
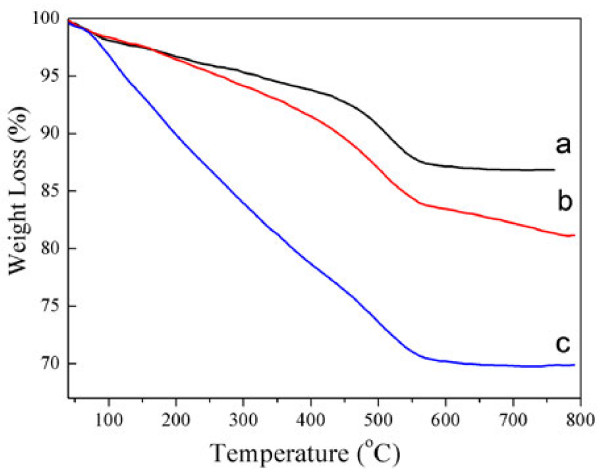
TGA of APTES-treated (a), azide-modified (b), click and ATRP-functionalized (c) silica nanoparticles.

The TGA curves of the functionalized nanoparticles are shown in Figure 6, from which an approximate amount of functional groups on the silica surface could be confirmed. The weight loss of APTES-treated SNPs was about 13%, which is attributed to the loss of the APTES layer. We can see obviously from curve b of Figure 6 that the weight loss of azide-modified nanoparticles is about 18.9% for the whole temperature range. It was calculated that there was about 5.9% mass loss for azide molecule-functionalized SNPs. After click chemistry and ATRP simultaneously, the first stage shows a weight loss of about 21% at 400°C while the second stage accounts for 8.7% of another weight loss (Figure 6c). These two stages of weight loss may indicate the degradation of PEGMA and that 1,2,3-triazole ring derivates molecules on the surface of SNPs, respectively.

**Figure 7:**
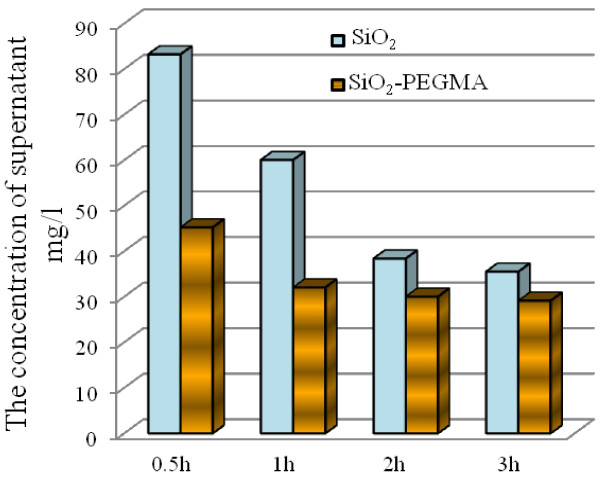
**The comparison of supernatants’ Pb**^**2+**^**concentration in SiO**_**2**_**and SiO**_**2**_**-PEGMA solutions.** The initial concentration of Pb^2+^ is 100 mg/l; the volume of Pb^2+^ is 10 ml; the weight of nanoparticles is 50 mg; the adsorption times are 0.5, 1, 2, and 3 h; and the temperature is 25°C.

The mechanism of metal ion adsorption from aqueous solution is related with physical and chemical processes, and the chemical binding reactions which happened involve metal ions and surface functional groups [36]; adsorption will be affected as the functional groups increase. After the successful preparation of PEGMA-modified SNPs, functionalized nanoparticles were utilized to absorb lead ions in the aqueous solution. From the adsorption experiment, the concentration of supernatant fluid is presented in Figure 7. We can find that polymerized SNPs were adsorbed faster than uncoated nanoparticles and the adsorption capacity of polymerized SNPs was also higher than that of uncoated nanoparticles, particularly within 1 h. It may be due to the polymerization process which can immobilize more functional groups on the surface of SNPs to chelate metal ions.

## Conclusions

After introducing amino groups onto silica surfaces, the ATRP initiator was successfully immobilized via click chemistry while poly(ethylene glycol) methacrylate chain-modified SNPs were obtained. This method takes advantage of click chemistry to optimize ATRP, which we have offered as a versatile pathway to functionalize SNPs. It will develop a series of desired functional groups and also extend this current different functional polymer chains which have potential application in the fabrication of SNP-based nanocomposites. Aside from the applications that require the enrichment of lead, because of the diversity and controllable ability of functional molecules on the surface of SNPs, efforts in our group are being made to extend current work to applications in selective removal of heavy metal ions such as Hg(II), Cu(II), As(III), and Cd(II).

## Competing interests

The authors declare that they have no competing interests.

## Authors’ contributions

WL carried out the preparation and modification of silica nanoparticles. YX participated in the analysis and the testing of the nanocomposite. YZ and WM supervised this work, helped in the analysis and interpretation of data, and, together with SW, worked on the drafting and revisions of the manuscript. WM and YD conceived of the study and participated in its design and coordination. SW participated in the design of the study and provided analysis instruments. All authors read and approved the final manuscript.

## Authors’ information

WL and YX are MD students. YZ is an associate professor and WM is a professor and the Dean of the Faculty of Metallurgical and Energy Engineering. SW is a postdoctoral fellow in Kunming University of Science and Technology. YD is an academician of the Chinese Academy of Engineering.
